# Genome-wide association study of Asian women identifies putative mammographic density-associated loci

**DOI:** 10.1186/s13058-025-02126-2

**Published:** 2025-11-21

**Authors:** Shivaani Mariapun, Mikael Eriksson, Mei-Chee Tai, Nur Aishah Mohd Taib, Cheng Har Yip, Kartini Rahmat, Celine M. Vachon, Sara Lindstrom, Jingmei Li, Mikael Hartman, Per Hall, Douglas F. Easton, Weang-Kee Ho, Soo-Hwang Teo

**Affiliations:** 1 Cancer Research Malaysia, Subang Jaya, Selangor Malaysia; 2 School of Mathematical Sciences, Faculty of Science and Engineering, University of Nottingham Malaysia, Semenyih, Selangor Malaysia; 3 Department of Medical Epidemiology and Biostatistics, Karolinska Institutet, Stockholm, Sweden; 4 Centre for Cancer Genetic Epidemiology, Department of Public Health and Primary Care, University of Cambridge, Cambridge, UK; 5 University Malaya Cancer Research Institute, Faculty of Medicine, University Malaya, Kuala Lumpur, Malaysia; 6 Department of Surgery, Faculty of Medicine, University of Malaya, Kuala Lumpur, Malaysia; 7 Subang Jaya Medical Centre, Subang Jaya, Malaysia; 8 Biomedical Imaging Department, Faculty of Medicine, University of Malaya, Kuala Lumpur, Malaysia; 9 Department of Quantitative Health Sciences, Mayo Clinic, Rochester, MN USA; 10 Department of Epidemiology, University of Washington, Seattle, WA USA; 11 Public Health Sciences Division, Fred Hutchinson Cancer Center, Seattle, WA USA; 12 Department of Surgery, Yong Loo Lin School of Medicine, National University of Singapore and National University Health System, Singapore, Singapore; 13 Genome Institute of Singapore, Agency for Science, Technology and Research (A*STAR), Singapore, Singapore; 14 National Cancer Center Singapore, SingHealth, Singapore, Singapore; 15 Saw Swee Hock School of Public Health, National University of Singapore and National University Health System, Singapore, Singapore; 16 Department of Oncology, Södersjukhuset, Stockholm, Sweden; 17 Centre for Cancer Genetic Epidemiology, Department of Oncology, University of Cambridge, Cambridge, United Kingdom

**Keywords:** Mammographic density, GWAS, Asian

## Abstract

**Background:**

Mammographic density (MD) is a strong, heritable risk factor for breast cancer. To date, 55 independent MD-associated genetic loci have been identified through genome-wide association studies (GWASs) in women of European ancestry; however, no studies have been reported in Asian women.

**Methods:**

To identify novel loci, we conducted genome-wide association studies (GWASs) of absolute dense, absolute nondense, and percentage area and volumetric densities, adjusting for age, body mass index (BMI), and ancestry-informative principal components, in a multi-ethnic cohort of 2,951 Asian women attending opportunistic mammography screening. We selected 175 novel loci that were associated with at least one MD phenotype at *P* < 5 × 10^− 6^ for (a) replication in an independent set of 401 Asian breast cancer cases, using density measurements from the unaffected breasts, (b) evaluation in a GWAS meta-analysis of MD in 27,900 women of European ancestry and (c) evaluation with breast cancer risk in Asian women.

**Results:**

Four of the 175 loci were replicated in women of Asian ancestry at *P* < 0.05, with directions of association that were consistent with those observed in the GWAS. The rs7018644 SNP at the 9p13.1 locus was the only loci replicated in both Asian and European cohorts. In addition, eight SNPs were also associated with breast cancer risk (*P* < 0.05) in a GWAS meta-analysis of Asian women.

**Conclusion:**

This study identifies potential novel MD-associated loci in Asian women. Replication in a larger Asian study is needed to confirm these findings.

**Supplementary Information:**

The online version contains supplementary material available at 10.1186/s13058-025-02126-2.

## Introduction

Mammographic density (MD), the absolute or relative amount of radiographically dense tissue, which represents epithelial or stromal breast tissue, is positively associated with breast cancer risk, where extensive density is associated with an increased risk of disease, while non-dense tissue, representing fatty tissue, is negatively associated [1–3]. There are differences in the distribution of MD between women of Asian and European ancestries, with Asian women having higher density on average, despite having lower age-standardised breast cancer incidence rates [4–8]. A large multiethnic study including over 866,000 women from eight states across the USA reported that Asian women had the highest prevalence of dense mammograms, defined as either heterogeneously dense or extremely dense by BI-RADS classification, among the ethnic groups studied (66.0% versus 45.5% in non-Hispanic White women, 45.3% in Hispanic women, and 37.0% in Black women) [9]. Regardless of the population differences in MD, a global study of MD (14,000 cases and 226,000 controls) showed that MD is equally predictive of risk to breast cancer across diverse populations [1–3, 10].

A significant proportion of the variability in MD can be attributed to heritable factors, with heritability estimates ranging from 60 to 75% [11–13]. Twin studies have demonstrated that the correlation of MD between monozygotic twins was double that of dizygotic twins, suggesting that alleles at multiple genetic loci are acting additively to influence MD. Genome-wide association studies (GWAS) in European women have identified 55 independent MD-associated loci, collectively accounting for more than 12% of its heritability [14, 15]. A recent study of multi-ethnic Asian women comprising participants of Chinese, Malay and Indian ancestries showed that only 35% of all known MD-associated variants identified in European populations were associated with MD in women of Asian ancestry [16]. Another recent GWAS conducted based on 1,333 women of African ancestry reported 31 candidate MD-regions at sub-genome-wide significance level (*P* < 5 × 10^− 5^), of which five were previously associated with breast cancer risk [17].

Given the differences in the genetic architecture across different populations, we hypothesise that a GWAS of MD in Asian women may uncover additional MD-associated loci. In this study, we conducted genome-wide association studies (GWASs) of area and volumetric density measurements derived using fully-automated methods in study populations of 2,450 Asian women for area-based densities, and 2,257 women for volumetric-based densities. SNPs showing suggestive associations with MD in the discovery analyses were further evaluated in independent cohorts of women of Asian and European ancestries.

## Methods

### Study participants and mammographic density measurement

The discovery cohort comprised of healthy women between the ages of 40 and 74 years who were participants of the Malaysian Mammography Study (MyMammo), an opportunistic screening program at two hospitals in Malaysia. The replication cohort consisted of mammograms of the unaffected breast performed not more than 12 months before diagnosis of breast cancer cases recruited into the Malaysian Breast Cancer Genetics Study (MyBrCa). The MyMammo and MyBrCa studies have been previously described [18]. All participants included in the study had raw and/or processed mammograms, and information on age and body mass index (BMI) at the time of mammography.

Full-field digital mammograms (FFDM) for 2,951 women in MyMammo and 401 women in MyBrCa, for which genome-wide genotyping was available, were obtained. Mammograms were performed on either Hologic, GE or Siemens mammogram systems. All measurements were conducted on the cranio-caudal (CC) view mammograms. MD estimation was obtained using STRATUS (version 1.8) [19] and Volpara (version 1.5.4) [20]. STRATUS analyses processed images and provides area-based estimates, whereas Volpara analyses raw images and provides volumetric density measurements respectively. In total, STRATUS measurements were available for 2,450 women in the discovery cohort and 378 women in the replication cohort, and Volpara measurements for 2,257 women in the discovery cohort and 314 women in the replication cohort. Measurements for both STRATUS and Volpara were available for 1,756 and 291 study participants in the discovery and replication cohorts, respectively. Six MD phenotypes were evaluated: absolute dense area (DA), non-dense area (NDA), percent dense area (PDA: dense area/total breast area) from STRATUS and dense volume (DV), non-dense volume (NDV) and percent dense volume (PDV: dense volume/total breast volume) from Volpara.

### Genotyping and imputation

All samples were genotyped using the Infinium OncoArray-500 K BeadChip (OncoArray) [21]. For the discovery cohort, samples were genotyped in two batches, while the replication cohort samples were genotyped in three batches. Genotyping, quality control and imputation were conducted by the central laboratory at the University of Cambridge as part of a larger breast cancer risk analysis [22]. The genotyping and quality control procedures used are as described previously [22]. Imputation was conducted using the 1000 Genomes Project (Phase 3) reference panel for all datasets. SNPs were excluded from further analyses if they had overall minor allele frequencies of < 0.01 in controls and imputation r^2^ < 0.3. All reported genomic positions are based on Genome Reference Consortium GRCh37 (hg19).

## Statistical methods

Box-Cox transformations were used to find the appropriate power transformation to apply to the MD phenotypes so the measurements approximated a normal distribution. Cube-root, square-root and 6th root transformations were used on STRATUS dense area, percent dense area and non-dense area measurements, respectively. Natural log transformations were used for Volpara dense volume and percent dense volume, and 4th root transformation for non-dense volume measurements. The associations between all eligible SNPs and transformed MD phenotypes were assessed, within each genotyping batch, using linear regression analysis, adjusting for age, BMI. The account for population structure, the analyses were also adjusted for the first 10 ancestry informative principal components estimated by genotyping batch (Figure S1). The association analysis were performed using SNPTEST (version 2.5.2) [23]. Meta-analyses across batches was conducted using METAL (version released on 2020-05-05) [24]. All statistical analyses were performed using R 3.6.3.

## Selection of potential novel SNPs

We considered SNPs with an association *P* value < 5 × 10^− 6^ for any of the six MD phenotypes as a potential MD-associated variant. Potential MD-associated SNPs which were not in linkage disequilibrium (LD) with previously reported MD-associated variants (r^2^ < 0.1) [15, 25–30] in the East and South Asian populations within the 1000 Genomes Project were considered potentially novel. In regions where multiple correlated SNPs (in LD with r^2^ > 0.1, within 200 kb of each other) were found to be associated with MD, the SNP with the smallest *P* value was selected for the replication study. The extents of the association signals were also visually inspected using Manhattan plots and regional association plots.

## Evaluation of potential novel SNPs in women of European ancestry

To evaluate the association of potential novel MD loci identified in this the Asian GWAS among women of European ancestry, we utilised summary statistics from a published GWAS meta-analysis of up to 27,900 women [26]. This meta-analysis included 30 studies in total, with GWAS conducted individually in 21 studies and subsequently meta-analysed alongside summary statistics from 9 previously published studies using a sample-size weighted approach [31]. For the 21 individual studies, linear regression was performed separately to assess the association between SNPs and square root–transformed MD phenotypes, adjusting for age, inverse BMI at mammography, and the first ten ancestry-informative principal components. The MD phenotypes used include dense area (*N* = 24,579), percent dense area (*N* = 27,900) and non-dense area (*N* = 24,689), all measured using CUMULUS, a semi-automated, reader-dependent computer-assisted thresholding method [31]. The genotype data were generated using the iCOGS, a custom Illumina Infinium array, and the Infinium OncoArray-500 K BeadChip (OncoArray) and the 1000 Genomes Project Phase 3 reference panel was used for genotype imputation [32]. SNPs with minor allele frequencies (MAFs) < 0.01 or imputation quality scores, r^2^ < 0.3, were excluded from the analyses.

### Association with breast cancer risk

To assess the associations of potential MD loci identified in this study with breast cancer risk in women of Asian ancestry, we utilised GWAS meta-analysis of breast cancer risk in Asian populations. This meta-analysis were conducted using summary statistics from three GWASs including (a) 8,245 invasive cases and 7,645 controls from Malaysia and Singapore (the controls in the Malaysian dataset overlapped with the samples in the MD GWAS), (b) 6,325 invasive cases and 73,225 controls from BioBank Japan, and (c) 19 studies participating in the Breast Cancer Association Consortium (BCAC) with a total of 10,355 cases and 10,189 controls (summary statistics were obtained from the BCAC website). GWASs for dataset (a) and (c) were conducted using logistic regression analyses adjusted for age and first two PCs while GWAS for dataset (b) was conducted using. Generalised linear mixed model, adjusted for age and first five principal components [33]. We used the METAL software (version released on 2020-05-05) to conduct fixed effects inverse variance meta-analysis of the three GWASs.

Descriptions of cohorts in the mammographic density GWAS discovery, replication and validation analyses, and the analysis of MD-associated variants with Asian breast cancer risk are provided in Table S1.

## Expression quantitative trait loci (eQTL) analyses

For MD-associated SNPs identified in this study that (a) were also associated with breast cancer risk and/or, (b) showed strong association with multiple MD phenotypes (*P* < 5 × 10^− 7^ ), we assessed the correlation between the genotype of these SNPs and gene expression in normal breast tissue and subcutaneous adipose tissue of individuals in the GTEx project (Data source: GTEx Analysis Release V10, dbGaP Accession phs000424.v10.p2) using eQTL analysis results within the GTEx database.

## Results

### GWAS of MD phenotypes

We conducted GWAS of area-based MD in 2,450 and volumetric MD in 2,257 Asian women, respectively. Of those, a total of 1,757 women had both area and volume-based measurements available. Characteristics of the included women are described in Table S2. Compared to those with volume-based density, women with area-based density were younger (52.7 vs. 54.0 years), had lower BMI (25.1 vs. 25.5 kg/m^2^), were more likely to be premenopausal (42.7 vs. 37.7%), Chinese (60.2 vs. 53.8%) and had mammograms from one of the hospitals (68.1 vs. 43.8%) and with a Hologic mammogram machine (95.4 vs. 83.8%), (*P* < 0.01; Table S2). No differences were observed for the parity-related variables between the two cohorts.

**Fig. 1:**
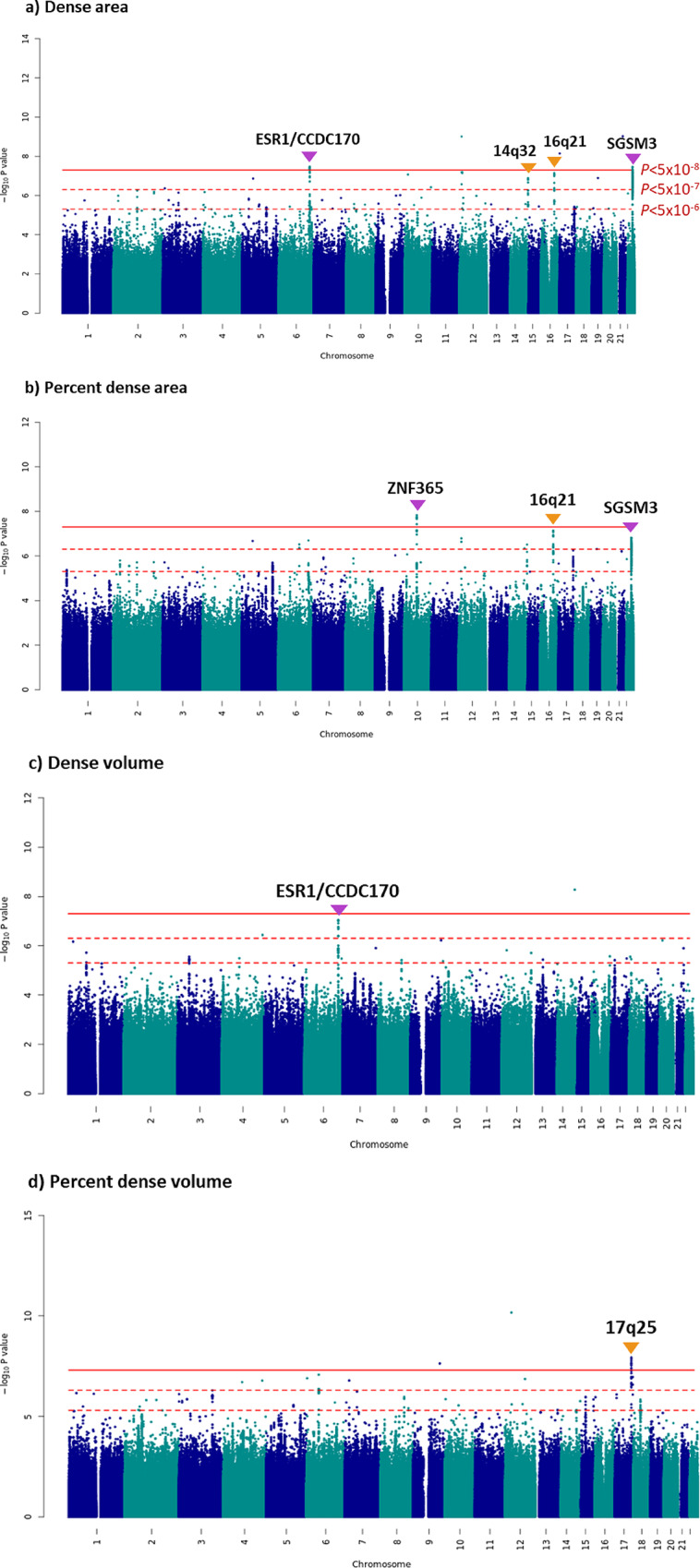
Manhattan plots from the GWAS of **a** Dense area, **b** Percent Dense Area, **c** Dense Volume and** d** Percent Dense Volume, in Asian women. Purple arrows indicate known mammographic density loci, orange arrows are used to annotate peaks at potentially novel density-associated loci

Six MD phenotypes (absolute area and dense volume, area and volumetric non-dense regions, and percent area and percent volumetric density) were evaluated. Strong correlations were observed for the left and right breast density measurements for STRATUS and for Volpara (Figure S2). As a slightly larger number of measurements were available from the left breast side mammograms, the MD measurements from this side were used in the discovery GWAS and the measurements from the unaffected breasts were used in the replication study. Association p-values for each of the MD phenotypes are shown in Figs. 1 and S4, and the corresponding quantile-quantile plots are shown in Figure S3.

We found strong signals, reaching the conventional GWAS significance threshold of *P* < 5 × 10^− 8^, at three regions that was previously identified in European women and validated in Asian women: 6q25.1 (dense area, *ESR1*), 10q21.2 (percent dense area, *ZNF365*), and 22q13.1 (dense area, *SGSM3*) (Table S3). In addition, we found 175 putatively novel independent genetic loci that were significantly associated with at least one MD phenotype at *P* < 5 × 10^− 6^ and were in low LD (r^2^ < 0.1) within 200 kb of any of the 64 previously reported MD-associated variants (within 55 independent loci) (Table S4). Of these, 8 SNPs (rs143085373, rs145100061, rs59337923, rs200042560, rs143801201, rs9893867, rs147583024 and rs1044228) reached the genome wide significance threshold of *P* < 5 × 10^− 8^ (Table S2).

### Replication in women of Asian ancestry

**Fig. 2:**
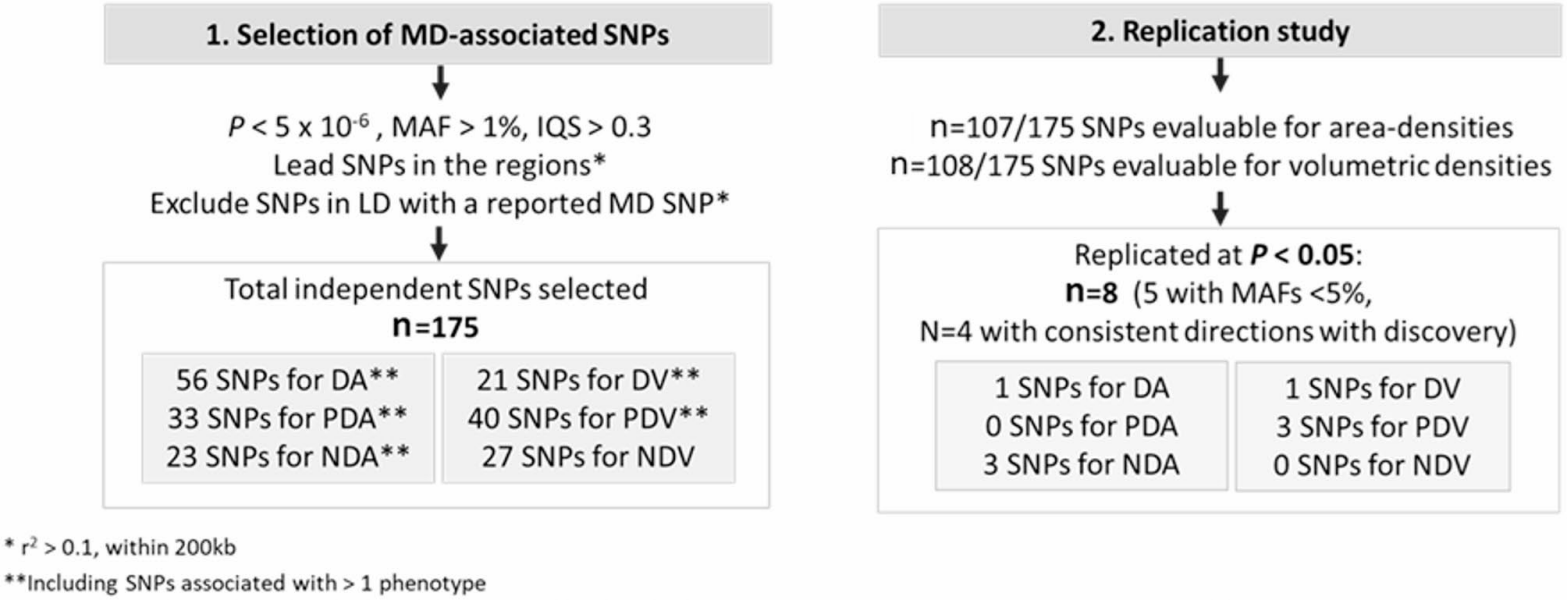
Summary of results from the GWAS and replication of MD in Asian women

**Fig. 3:**
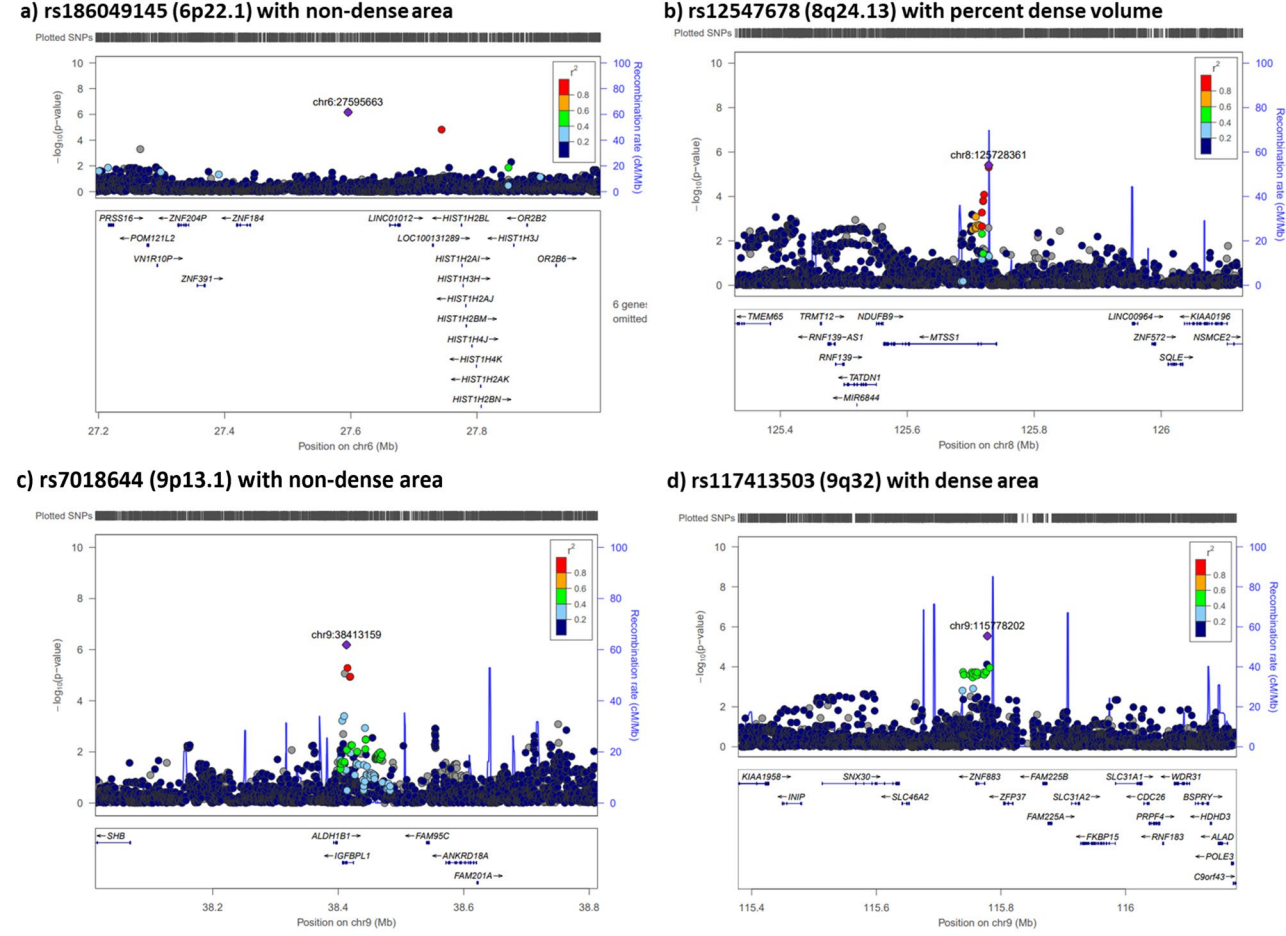
Regional association plots for GWAS hits replicated at consistent directions of association with unaffected breasts from Asian breast cancer cases **a)** rs186049145 (6p22.1), **b)** rs12547678 (8q24.13, *MTSS1*), **c)** rs7018644 (9p13.1, *IGFBPL1*) and **d)** rs117413503 (9q32)

**Fig. 4:**
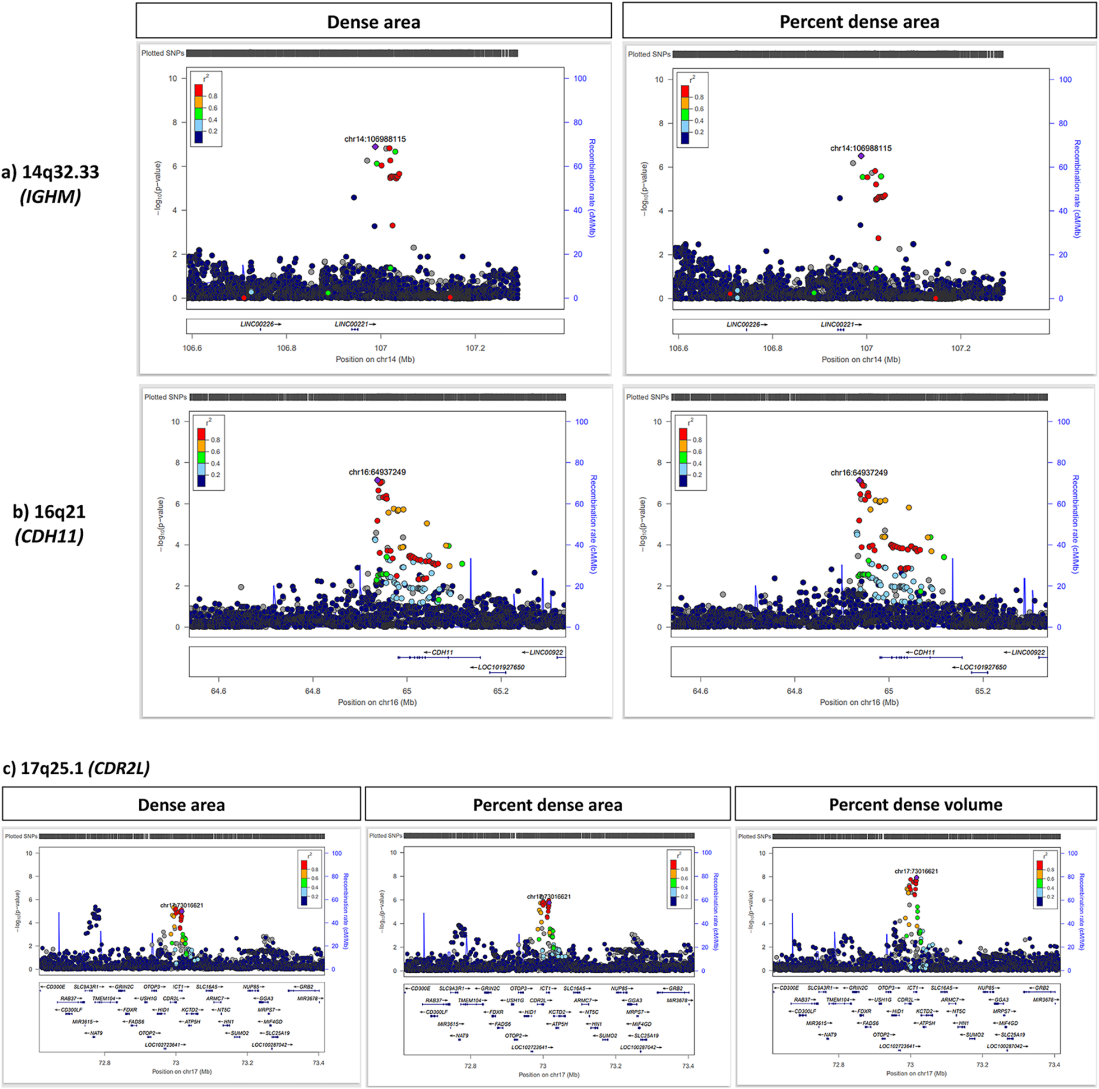
Regional association plots for top GWAS hits **a)** 14q32.33 (*IGHM*),** b)** 16q21 (*CDH11*) and** c)** 17q25.1 (*CDR2L*)

Of the 175 SNPs, 107 and 108 SNPs had MAF > 0.01 and imputation quality score > 0.3, and were evaluated in the area-based and volumetric density replication cohorts of Asian ancestry, respectively (Fig. 2). Eight SNPs were replicated at *P* < 0.05, of which only 4 (6p22.1, 8q24.13, 9p13.1, and 9q32) showed a consistent direction of association with the discovery study (Table 1). The strongest signal was rs186049145 on 6p22.1 where *P* = 6.8 × 10^− 7^ in the discovery GWAS and *P* = 6.3 × 10^− 6^ in the replication analyses. Regional association in Fig. 3(a) for the 6p22.1 region showed that there was only one correlated neighbouring SNP in this region. The regional association plots of the remaining 3 loci (8q24.13, 9p13.1 and 9q32) show multiple SNPs in LD with the lead SNP which had associations that were significant at *P* < 5 × 10^− 4^ (Fig. 3). The corresponding *P* values in replication analyses for these three regions were 0.024, 0.007 and 0.046, respectively. Three of the 175 loci, namely 14q32.33 (*IGHM*), 16q21 (*CDH11*) and 17q25.1 (*MRPL58*), had multiple correlated SNPs having significant associations at *P* < 5 × 10^− 7^ and were also found to be associated with multiple area or percent MD phenotypes (Figs. 1 and 4), however, none of which could be replicated in the replication dataset (Table S5).

**Table 1:**
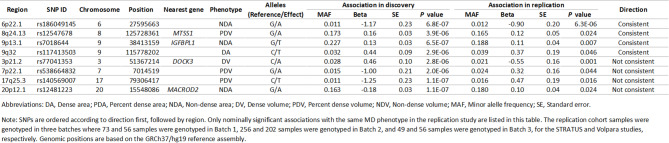
Potential novel mammographic density (MD) associated loci identified in Asian women that are associated with MD at *P*<0.05 in the replication study

### Evaluation in women of European ancestry

**Fig. 5:**
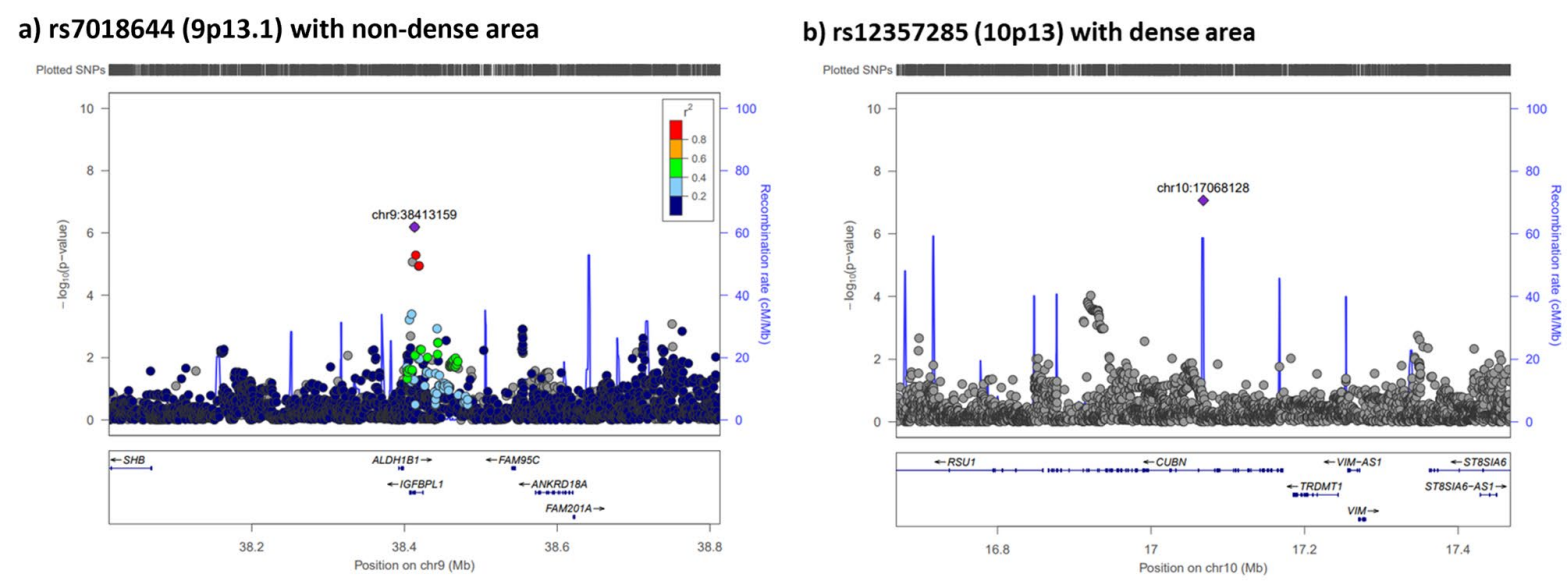
Regional association plots for GWAS hits validated at consistent directions of association with women of European ancestry a) rs7018644 (9p13.1, *IGFBPL1*) and b) rs12357285 (10p13, *CUBN*).

We examined the associations of the 175 potential novel MD-associated SNPs with area-based measurements of MD in a set of 27,900 women of European ancestry. Of these 175 SNPs, 83 (47.4%) had MAF > 0.01 and imputation quality score > 0.3 in European women and could be evaluated. Significant associations at *P* < 0.05 were observed for four SNPs (rs7018644, rs12357285, rs72964043, and rs7175269), of which two SNPs (rs7018644 at 9p13.1 and rs12357285 at 10p13) showed directions of associations that were consistent with our findings in the discovery analyses (Table 2; Fig. 5). Based on the standardised effect estimates (Z-scores in Table 2), the magnitude of association for these two SNPs was smaller in the European women study. One of these two SNPs (rs7018644 at 9p13.1) was also one of the four SNPS replicated in the Asian replication analyses. Of the remaining three regions replicated in the Asian analyses (6p22.1, 8q24.13, 9q32), two were not evaluable in European women (6p22.1 and 9q32), and one (8q24.13) had a magnitude of association that was smaller and direction of association that was opposite in women of European ancestry (data not shown).

**Table 2:**
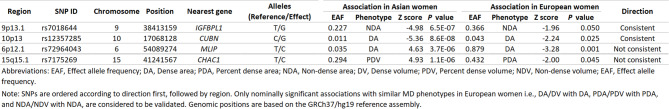
Potential novel mammographic density (MD) associated loci identified in Asian women that are associated with MD at *P*<0.05 in women of European ancestry

### Association with breast cancer risk

Of the 175 selected SNPs, 114 had association statistics for breast cancer risk, based on the meta-analysis of 24,925 and 91,059 Asian breast cancer cases and controls. Eight out of the 114 SNPs (rs146050480, rs3806759, rs114542182, rs77041353, rs279075, rs1356093631, rs149134505, and rs12599695) were significantly associated with breast cancer risk at *P* < 0.05 (Table 3). Three of these were associated with both dense area and percent dense area, while five SNPs were associated with volumetric density phenotypes, namely dense volume (*n* = 2), percent dense volume (*n* = 1) and non-dense volume (*n* = 2). Four of the eight SNPs associated with risk are monomorphic in the 1000 Genomes European and South Asian populations, and one is very rare (MAF = 0.004) in East Asians. Out of the eight SNPs, six showed directions of the breast cancer association that were consistent with that observed for MD (rs146050480, rs3806759, rs77041353, rs279075, rs1356093631 and rs149134505). None of the eight SNPs associated with breast cancer risk in the Asian meta-analysis at *P* < 0.05 have previously been associated with breast cancer risk.

**Table 3:**
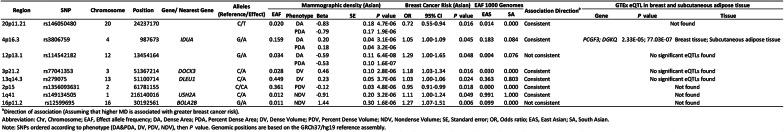
SNP’s associated with volumetric and area densities at *p* < 5 × 10^-6^ that are also significantly associated with breast cancer risk in Asian women at *P* < 0.05

### Expression quantitative trait loci (eQTL) analyses

For the eight candidate MD-SNP that were associated with MD and breast cancer risk in Asian women, we examined the correlation between genotype and gene expression in normal breast tissue using eQTL analyses data in the GTEx database (Table 3, Figure S5). The eQTL of one out of the eight SNPs evaluated, rs3806759, showed significant associations for the expression levels of (a) *PCGF3* in breast tissue and (b) *DGKQ* in subcutaneous adipose tissue [34]. Given the limitations in the sample size for the replication analysis, we considered the possibility that SNPs that were associated with multiple MD features at *P* < 5 × 10^− 7^ may be true positives that were not replicated because of sample size. Indeed, eQTL analyses for the three loci (14q32.33, 16q21 and 17q25.1) were not significant for breast tissue, but two SNPs, rs11646481 and rs1044228, had significant associations for the expression of *ENSG00000278893* and HID1, respectively, in subcutaneous adipose tissue (Table S5, Figure S5).

## Discussion

In this GWAS of area and volumetric MD in Asian women, we found 175 potential novel loci that were significantly associated with at least one MD phenotype at *P* < 5 × 10^− 6^. Four loci (6p22.1, 8q24.13, 9p13.1 and 9q32) were replicable at the nominal significance level (*P* < 0.05) in our replication study of unaffected breasts of Asian breast cancer cases. Only 83 out of the 175 loci (47%) could be evaluated in European women, and the remaining 53% were either monomorphic or extremely rare. Notably, more than 75% (134 SNPs) of the 175 loci had minor allele frequencies that were between 1 and 5%. Of these 83 SNPs, we found that four loci were significantly associated (*P* < 0.05) with MD, and of those only two, rs7018644 at 9p13.1 and rs12357285 at 10p13, had directions of associations that were consistent with that observed in our Asian study. Additionally, eight (4.6%) of the potentially novel MD SNPs, namely rs146050480, rs3806759, rs114542182, rs77041353, rs279075, rs1356093631, rs149134505, and rs12599695, were significantly associated with breast cancer risk in Asian women.

Two SNPs were identified in our analysis (rs7018644 at 9p13.1 and rs12357285 at 10p13) and showed nominal association with MD in women of European descent. The rs7018644 SNP at the 9p13.1 locus in the intronic region of *IGFBPL1* was associated with non-dense area and was the only variant in our GWAS that showed consistent replication in both Asian breast cancer cases and the validation study in women of European ancestry. While the functional consequence of rs7018644 is currently unknown and we did not find direct evidence linking this SNP to altered *IGFBPL1* expression or to breast cancer risk, we note that IGFBPL1 belongs to insulin-like growth factor binding proteins (IGFBPs) family, which are known to regulate the functions of insulin-like growth factors. IGFBPL1 was reported to play a role in neural development and notably, its expression is reduced in breast cancer, potentially due to aberrant hypermethylation of its promoter region [35, 36]. Although these observations provide biological context for the gene, further functional analyses are required to determine whether rs7018644 or variants in linkage disequilibrium (LD) with it exert any regulatory effect on *IGFBPL1* or other nearby genes.

The rs12357285 SNP at 10p13, is an intronic variant in *CUBN* and was associated with MD in our discovery dataset and European women, but not validated in our dataset of unaffected breasts in breast cancer cases. Notably, rs12357285 is monomorphic in the East Asian population, suggesting further analyses in other populations may be helpful.

Of the 8 candidate MD-SNP that were associated with both mammographic density and breast cancer risk in this Asian study, one (rs3806759) showed significant associations with the expression of *PCGF3* in breast tissue and *DGKQ* in subcutaneous adipose tissue. Additionally, among the three loci strongly associated with multiple MD phenotypes in the discovery analysis but not replicated and also not associated with breast cancer risk, two SNPs (rs11646481 and rs1044228) were associated with gene expression in adipose tissue. Although these findings are preliminary, they suggest that some MD-associated variants may influence mammographic traits through effects on gene expression in adipose or other relevant tissues. These results support the need for further functional follow-up to clarify the biological pathways linking these variants to mammographic density and breast cancer risk.

Our study highlights the value of conducting GWAS in diverse Asian populations, as variants rare or monomorphic in one population may be more frequent in others, thereby improving the power to uncover novel loci relevant to specific ancestral groups. One such example is the rs186049145 SNP at 6p22.1 which was found to be associated with non-dense tissue in both the discovery (*P* = 6.8 × 10^− 7^) and Asian replication study (*P* = 6.3 × 10^− 6^), and had only one correlated neighbouring SNP (rs181589717, imputation quality score = 0.63) showing similar strengths of association as the lead SNP. Notably, rs186049145 is monomorphic in Europeans, Africans and South Asians in the 1000 Genomes Project and is very rare (MAF = 0.005) in East Asians, and no association with NDA has previously been reported in this region. However, in our multi-ethnic GWAS of Chinese, Malay and Indian women, the minor allele frequency was higher (MAF = 0.011 overall), enabling the detection of this association.

While GWAS of mammographic density in European women have identified 55 independent loci, these variants collectively explain only a modest proportion (~ 12%) of its heritability, indicating that additional genetic factors remain undiscovered. More recently, a MD GWAS in 7,040 women of European ancestry which utilised mammographic texture variation features instead of conventional MD phenotypes, identified three loci that have not previously been associated with MD or breast cancer risk [37]. Notably, none of the 175 MD-associated loci identified in our study were correlated with loci reported previously in the European or African GWASs [15, 17, 25–30]. Our findings suggest that genetic determinants of MD may differ across populations, as most loci identified in European populations were not replicated in Asian populations as shown in our previous study [16], and conversely, the loci identified in our Asian cohort were not replicated in Europeans as shown in this study. These differences highlight the importance of conducting ancestry-specific studies to comprehensively map MD-associated variants and improve our understanding of the genetic architecture underlying MD. Larger studies in diverse populations are needed to fully capture the heritability of MD and assess whether population-specific or shared genetic factors contribute to MD and breast cancer risk.

Our study has several limitations. Firstly, the relatively small sample size in the replication cohort may have limited power to validate associations observed in the discovery analysis, especially for variants with modest effect sizes or lower allele frequencies. Secondly, the possibility of false positive findings. For the purpose of identifying a larger number of potential MD-associated loci to take forward into the replication step, we lowered the conventional GWAS threshold for statistical significance to *P* < 5 × 10^− 6^. This increases the risk of false positive findings in the discovery step, and may also in part explain the lack of replicability observed in this study. Additionally, while the evaluation of potential MD-associated loci with breast cancer risk helps to prioritise potential novel MD loci with functional relevance, we acknowledge the possibility of false positive findings. Given that MD and breast cancer, though correlated, have distinct genetic architectures, some associations may arise due to chance rather than true biological effects. The single-variant lookup approach using the lead SNPs identified in the GWAS for the eQTL analysis instead of performing a colocalization analysis using eQTL and GWAS summary statistics may have also resulted in false positive findings, i.e., incorrect genes identified for forming a causal hypothesis. Thirdly, different MD measurement methods were used for the European and Asian MD GWAS. It is therefore not possible to determine whether the lack of consistent associations in Europeans versus Asians is because of differences between populations or between the methods used, i.e., Cumulus and STRATUS respectively. Another consideration is the multiethnic study design. While it has provided us with a unique opportunity to identify MD-associated loci in understudied ethnic groups, this may have resulted in weaker associations at several loci due to genetic heterogeneity across the different ethnic groups. Studies have indicated that including participants from multiple ancestries may increase the number of genome-wide significant associations identified. However, given the lack of replicability, this study may have benefited from analysis methods that are better suited for a trans-ancestry GWAS such as a cross-ancestry meta-analysis of ancestry-stratified mixed effects GWAS.

Nevertheless, the key strength of this study is the inclusion of populations that have not been well studied previously, allowing us to interrogate alleles that are more common in non-European populations. Notably, of the 92 SNPs that could not be evaluated in European studies, 88 (95.7%) had MAFs that were below 1% in the European population, of which 73 (83.0%) were monoallelic. These SNPs could be included in our multi-ethnic GWAS of Malay, Chinese and Indian women because the minor alleles were more common, with frequencies of greater than 1% in the East and/or South Asian populations. These population-specific MD-associated alleles may provide insights into the ethnic differences seen for MD.

## Conclusion

In this GWAS of area and volumetric MD phenotypes in Asian women, putative novel MD-associated loci were identified and require further evaluation in a large, independent Asian cohort with greater representation of Southeast Asian and South Asian ancestries. Overall, this study demonstrates the value of conducting GWASs in diverse populations in adding to the knowledge of genetic contributors of inherited phenotypes. It is highly likely that at least some of the population differences in risk and MD is due to genetic factors and the findings of this study presents us with an opportunity to explore potential population-specific candidate genes. Functional studies will be necessary to map out the genes in potentially novel regions associated with MD. If we can establish that these genes also influence breast cancer risk in the population, we may open up possibilities for new targets for cancer prevention and risk prediction in the future.

## Supplementary Information

Below is the link to the electronic supplementary material.

Supplementary Material 1.

Supplementary Material 2.

## Data Availability

Summary statistics for all SNPs reported in this manuscript are provided in the Supplementary Table S4. Request for access to individual level data on which these analyses were based can be made via the corresponding author. Summary statistics for the mammographic density GWAS in women of European ancestry and for the breast cancer GWASs of Asian and European women within The Breast Cancer Association Consortium (BCAC) can be accessed from the BCAC website at https://www.ccge.medschl.cam.ac.uk/breast-cancer-association-consortium-bcac/data-data-access/summary-results.Summary statistics BioBank Japan (BBJ) are publicly available from the BBJ website at https://biobankjp.org/en/researchers/2013#gsc.tab=0.
