# Supplementary material for: Genome-wide association study of Asian women identifies putative mammographic density-associated loci
Source: Breast Cancer Res. 2025 Nov 21;27:207. doi: 10.1186/s13058-025-02126-2 (PMC12639730; doi:10.1186/s13058-025-02126-2)
Supplement: Supplementary file 2 — Supplementary Material 2. [file 13058_2025_2126_MOESM2_ESM.docx]

**b)**

**a)**


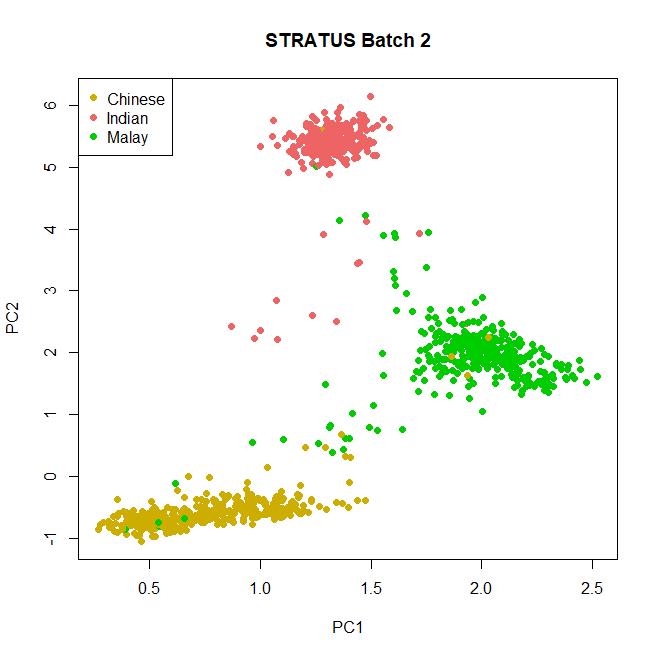

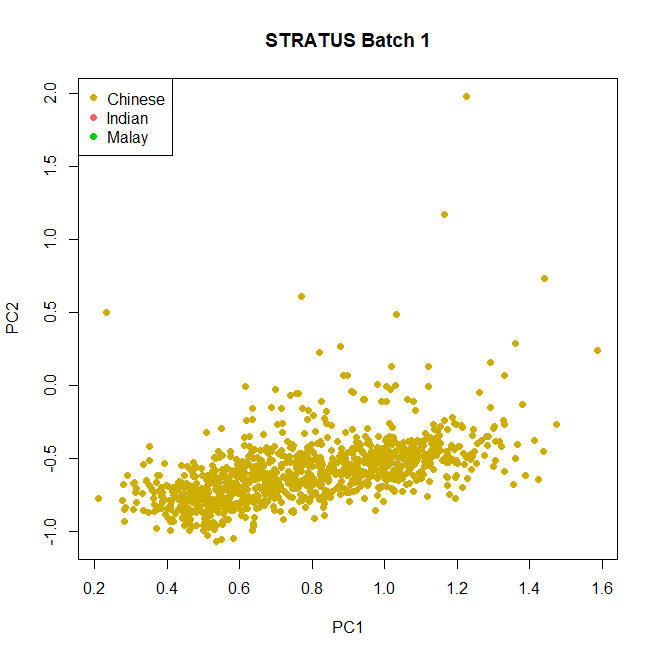

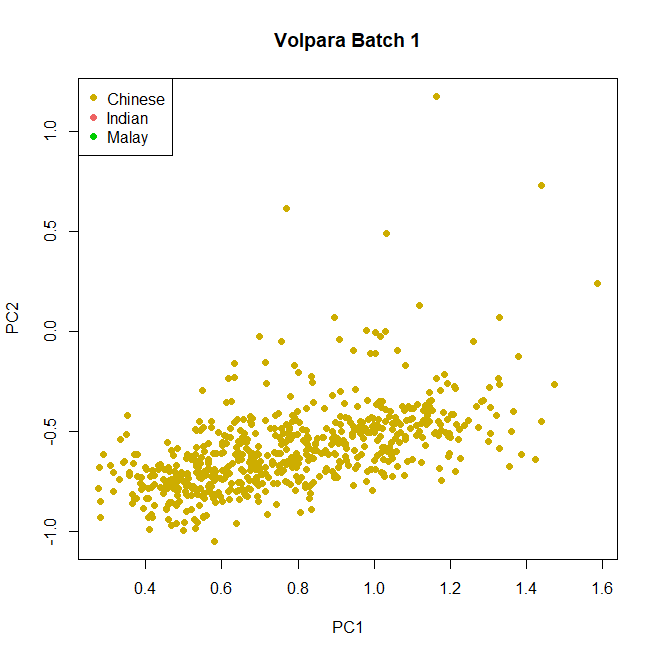

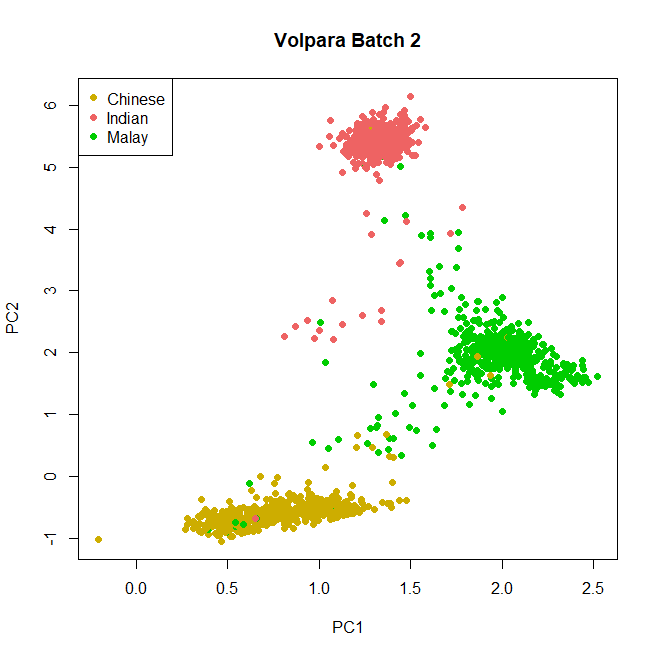


**c)**

**d)**

**Figure S1:** Ethnicity clustering, colour-coded according to self-reported ethnicity, using the first and second ancestry-informative principal components, PC1 and PC2, for samples used in the GWAS of **a)** STRATUS densities for samples genotyped in batch 1, **b)** Volpara densities for samples genotyped in batch 1, **c)** STRATUS densities for samples genotyped in batch 2, and, **d)** Volpara densities for samples genotyped in batch 2.


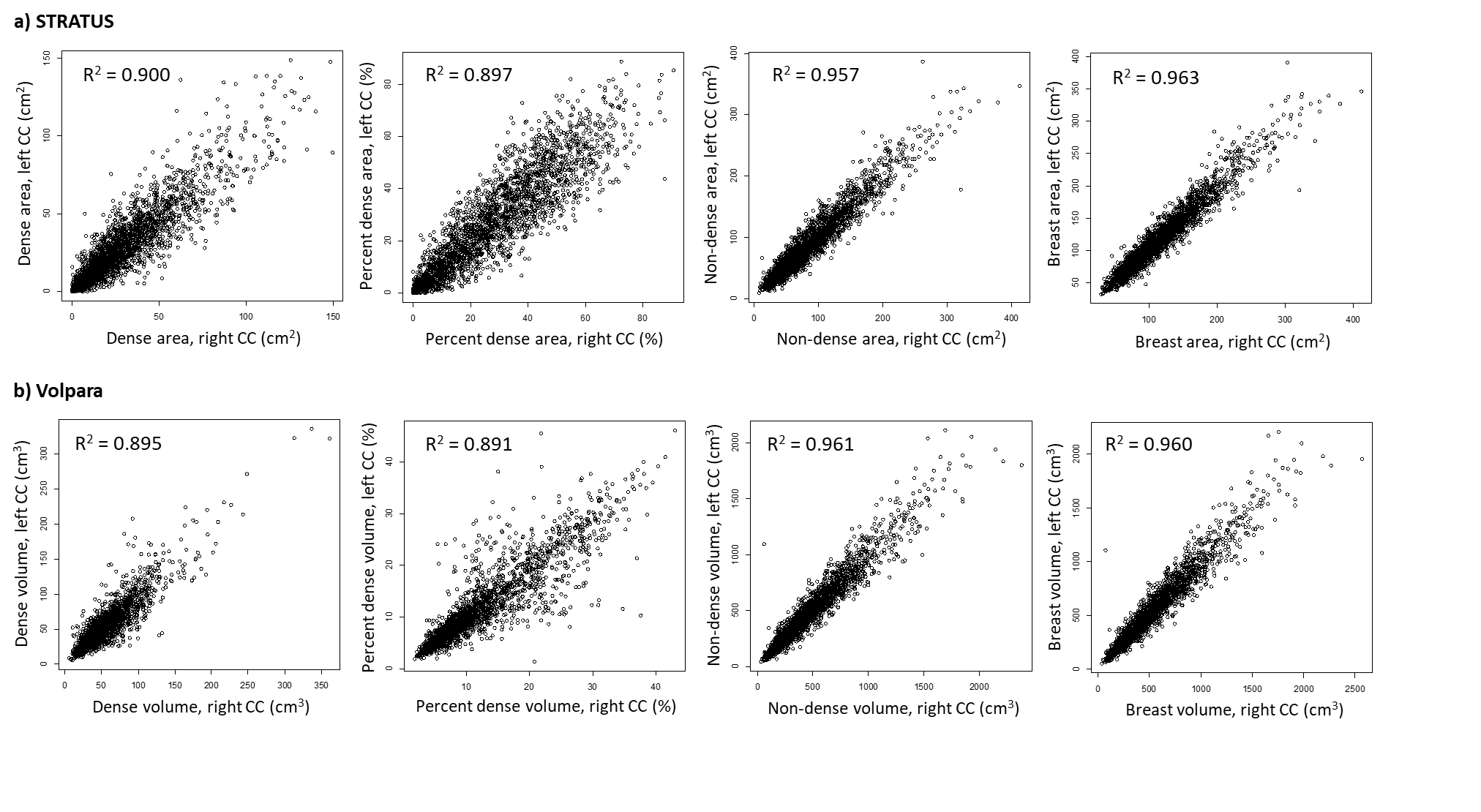


**Figure S2.** Scatter plots of left CC versus right CC view mammograms for **a)** STRATUS and **b)** Volpara measurements.


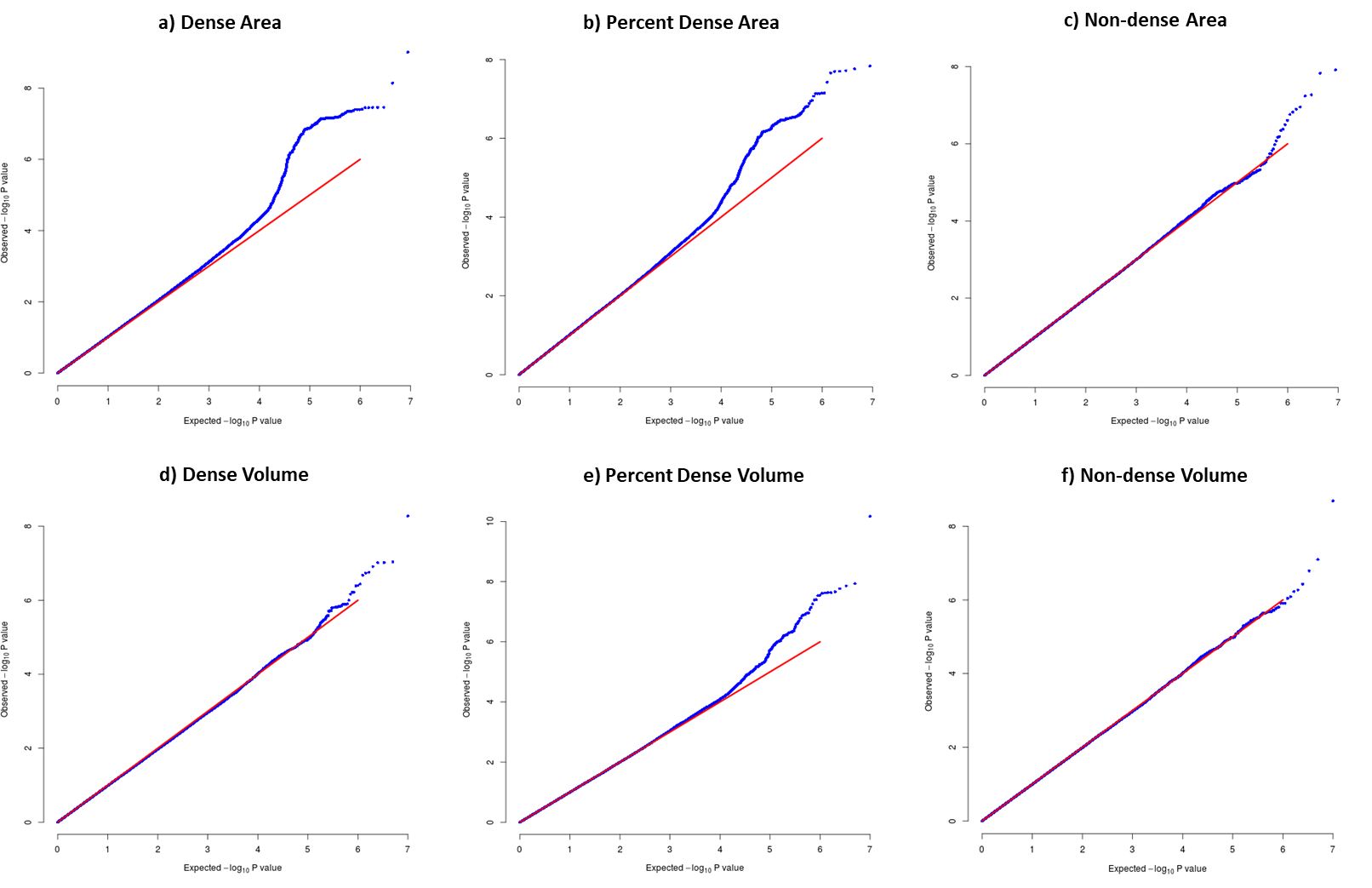


λ = 1.00

λ = 0.99

λ = 0.98

λ = 0.99

λ = 1.02

λ = 1.02

**Figure S3:** Quantile-quantile plots from the GWAS of a) Dense area, b) Percent dense area, c) Non-dense area, d) Dense volume, e) Percent dense volume and f) Non-dense volume, in Asian women. Note: λ = genomic inflation factor lambda for GWAS.


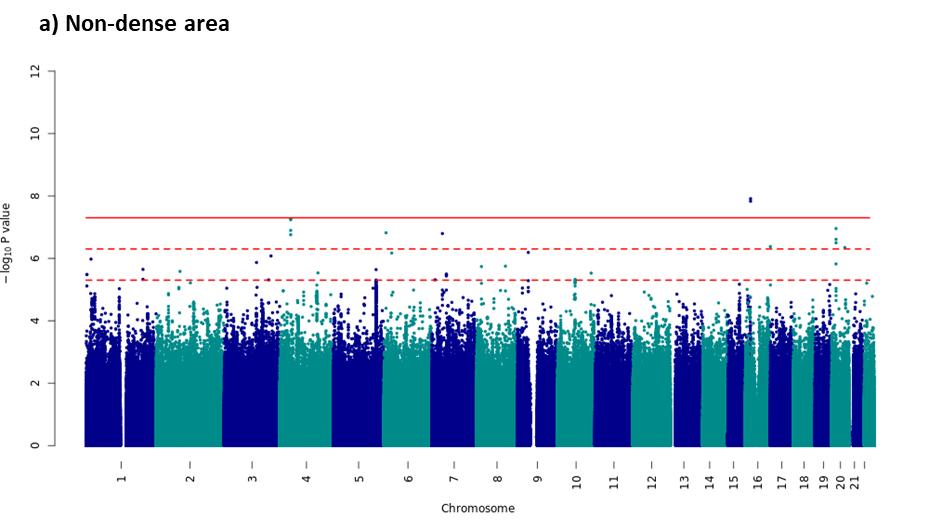


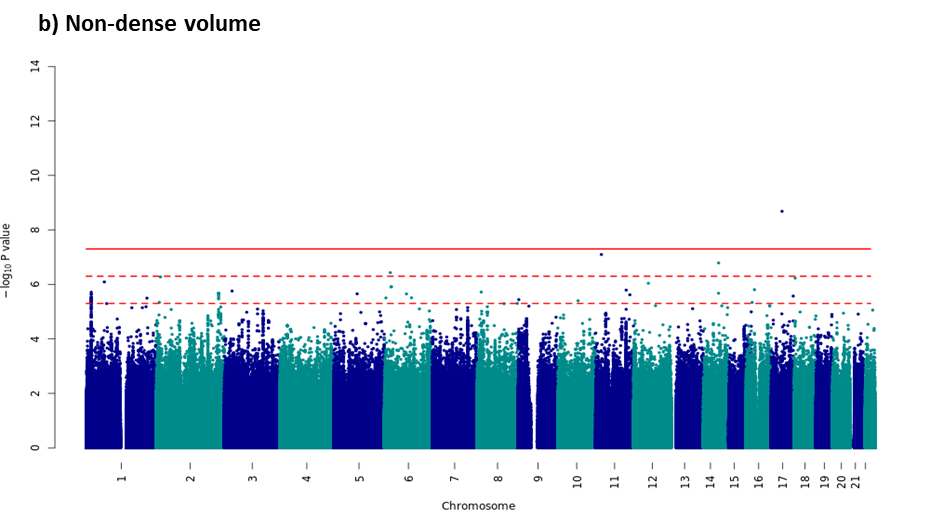


**Figure S4:** Manhattan plots from the GWAS of a) Non-dense Area and b) Non-dense Volume, in Asian women


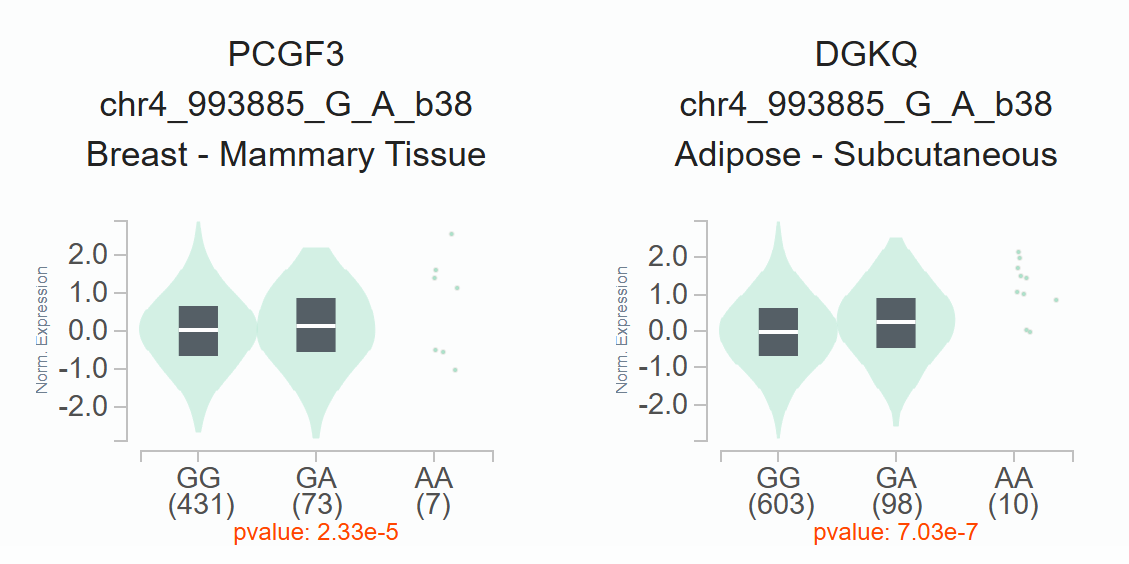


d)

c)


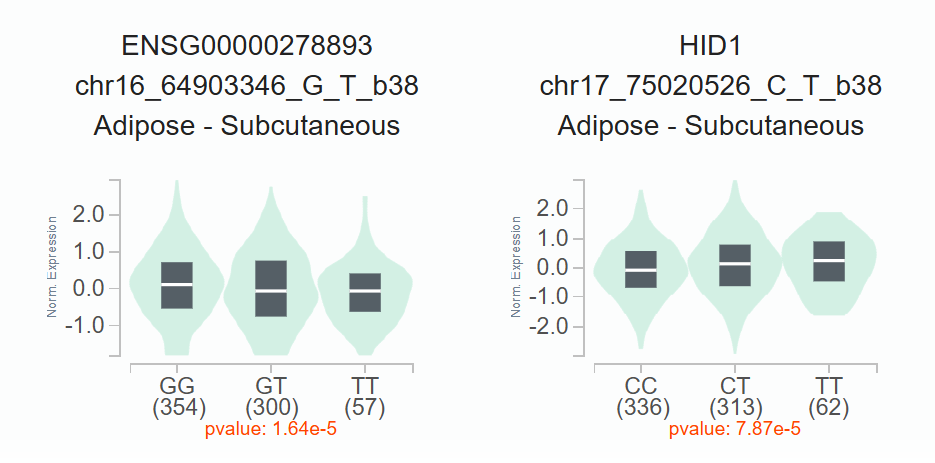


**Figure S5:** Violin plots for the eQTLs of rs3806759 with significant associations for the expression levels of a) *PCGF3* in normal breast tissue and b) *DGKQ* in subcutaneous adipose tissue, c) the eQTL of rs11646481 with a significant association for the expression of *ENSG00000278893* in subcutaneous adipose tissue, and d) the eQTL of rs1044228 with a significant association for the expression of *HID1* in subcutaneous adipose tissue. Data source: GTEx Analysis Release V10 (dbGaP Accession phs000424.v10.p2).
